# Effect of Hindwings on the Aerodynamics and Passive Dynamic Stability of a Hovering Hawkmoth

**DOI:** 10.3390/biomimetics8080578

**Published:** 2023-12-01

**Authors:** Ryusuke Noda, Toshiyuki Nakata, Hao Liu

**Affiliations:** 1 Department of Mechanical Engineering, Tokyo University of Technology, 1404-1 Katakura-cho, Hachioji 192-0982, Japan; 2 Graduate School of Engineering, Chiba University, 1-33 Yayoi-cho, Inage-ku, Chiba 263-8522, Japan

**Keywords:** insect flight, flapping wing, computational fluid dynamics, aerodynamics, dynamic stability, hindwing-less, hawkmoth

## Abstract

Insects are able to fly stably in the complex environment of the various gusts that occur in nature. In addition, many insects suffer wing damage in their lives, but many species of insects are capable of flying without their hindwings. Here, we evaluated the effect of hindwings on aerodynamics using a Navier–Stokes-based numerical model, and then the passive dynamic stability was evaluated by coupling the equation of motion in three degrees of freedom with the aerodynamic forces estimated by the CFD solver under large and small perturbation conditions. In terms of aerodynamic effects, the presence of the hindwings slightly reduces the efficiency for lift generation but enhances the partial LEV circulation and increases the downwash around the wing root. In terms of thrust, increasing the wing area around the hindwing region increases the thrust, and the relationship is almost proportional at the cycle-averaged value. The passive dynamic stability was not clearly affected by the presence of the hindwings, but the stability was slightly improved depending on the perturbation direction. These results may be useful for the integrated design of wing geometry and flight control systems in the development of flapping-winged micro air vehicles.

## 1. Introduction

The application of flying robots such as drones has spread rapidly in recent years [1]. The development of processing and control technologies and the miniaturization of devices have enabled stable flight even with small airframes. However, drones still face many challenges in terms of efficiency, stability, and safety. Drones are about the same size as insects and birds, and insects and birds can be the inspiration for drones, as they have achieved high-performance flight that outperforms drones [2].

Insects generate aerodynamic forces to support their own weight and adjust their size by means of flapping wings. The aerodynamic forces to support their own weight and adjust their posture are generated by unsteady aerodynamic mechanisms such as leading-edge vortices and rotational lift [3,4,5,6]. Since the unsteady aerodynamic forces are generated by the interaction between the flapping wing and the air, the wing morphology and kinematics have a significant influence on the aerodynamic performance of a flapping wing [7]. In particular, the effect of wing morphology and kinematics on efficiency has been extensively studied experimentally and theoretically [8,9,10,11,12].

In both flying organisms and robots, flight stability is very important, along with efficiency in generating aerodynamic forces. In insects, for example, attitude stabilization is achieved by adjusting wing motion [13]. They need to control their attitude with wing motions in response to their own attitude. On the other hand, there is a reaction delay of several flapping cycles, which significantly affects their flight performance [14]. The mechanical stability of insect flight is also very important from the point of view of efficiency, etc., because in nature there are unpredictable disturbances, such as wind, and if the posture is significantly disrupted during the sensory delay, more energy will be consumed to restore the posture. 

The mechanical stability of insect flight has been investigated by wind tunnel experiments and numerical simulations [15,16,17,18,19]. For example, numerical simulations have investigated the flight stability of various insects and confirmed that insects are unstable in the pitch and roll directions. Since stability and maneuverability are trade-offs, these results suggest that maneuverability is important for the insect’s habitat along with flight stability.

Thus, while the flight stability of insects has been investigated so far, the effect of wing morphology on flight stability has received little attention compared with its effect on efficiency. As the balance between stability and maneuverability is considered important in flying robots for various missions, it would be very important to derive design guidelines for flapping wings from the perspective of flight stability when creating bio-inspired flying robots.

In the present study, we focused on the effect of the presence of hindwings on flight stability, with particular emphasis on the hawkmoth as the target insect. It has been reported that lepidopterans can fly even when their hindwings are ablated [20]. This is likely due to the very small contribution of the hindwings to the generation of vertical forces, as aerodynamic forces are generated during wing flapping, especially near the wing tips. On the other hand, the effect of wing morphology on flight stability is unknown, as is the effect of hindwing ablation on flight stability. Therefore, in the present study, along with a model of the hawkmoth, a model of the hawkmoth with its hindwings removed was also created, and these models were used to investigate the effect of wing morphology, especially near the base, on the flight stability of the flapping wing by the numerical simulation. It is hoped that clarifying the effects of insect wing shape on stability will help to provide design guidelines for future wing shapes of flapping-winged micro air vehicles (FMAVs).

## 2. Materials and Methods

### 2.1. Morphological and Kinematic Model for a Hawkmoth with/without Hindwings

**Figure 1:**
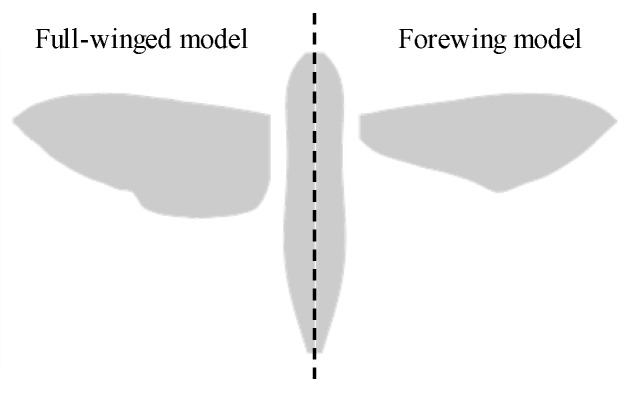
Morphological models with/without hindwings.

**Figure 2:**
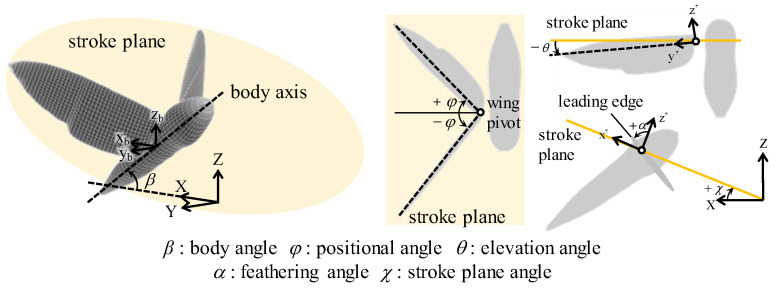
The definition of the coordinates and the kinematic angles.

**Figure 3:**
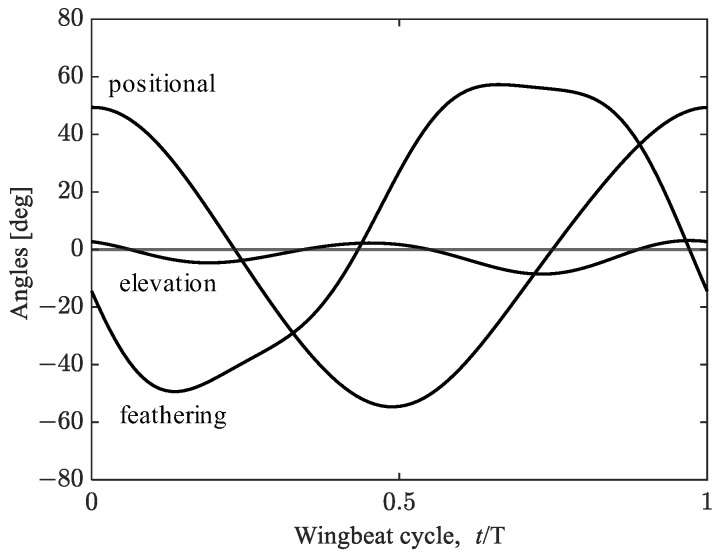
Wing kinematics of a hovering hawkmoth. *t*/*T* is the dimensionless time, where *T* is the period and *t* = 0 at the beginning of the downstroke.

A morphological and kinematic model of a hovering hawkmoth, *Agrius convoluvuli*, was built based on an experimental observation that was filmed with five synchronized high-speed cameras (Fastcam SA-3, Photron, Tokyo, Japan), and a three-dimensional reconstruction was performed with a direct linear transformation (DLT) method via the open-source MATLAB-based application, DLTdv5 [21]. More details can be found in [22]. The reconstructed morphological model with/without hindwings is depicted in Figure 1, and the morphological parameters are summarized in Table 1. The body orientation and flapping wing kinematics are described with the help of the global (X-Y-Z) and wing-fixed (x’-y’-z’) coordinate systems (Figure 2). The body-fixed coordinate system (x_b_-y_b_-z_b_) is defined for the dynamic analysis and is centered on the center of gravity. The hovering kinematic model is defined by the three angles expressed as the third-order Fourier series with respect to the stroke plane (Figure 3).

### 2.2. Numerical Model for Flow Field around a Flyer

For the analysis of the flow field around the hawkmoth model, we used a CFD solver based on a finite volume method and a fortified Navier–Stokes solver for a multi-blocked overset grid system [23]. The governing equations of the numerical solver are the three-dimensional incompressible unsteady Navier–Stokes equations written in strong conservation form for mass and momentum. The artificial compressibility method is used by adding a pseudo-time derivative of pressure to the equation of continuity. For an arbitrary deformable control volume *V(t)*, the non-dimensionalized governing equations are

(1) $$\int_{V\left(t\right)}\left(\frac{\partial Q}{\partial t}+\frac{\partial q}{\partial \tau }\right)dV+\int_{V\left(t\right)}\left(\frac{\partial F}{\partial x}+\frac{\partial G}{\partial y}+\frac{\partial H}{\partial z}+\frac{\partial F_{v}}{\partial x}+\frac{\partial G_{v}}{\partial y}+\frac{\partial H_{v}}{\partial z}\right)dV=0,$$

where bold letters are used to denote matrices as

Q=uvw0,q=uvwp,F=u2+puvuwλu,G=vuv2+pvwλv,H=wuwvw2+pλw,Fv=−1Re2uxuy+vxuz+wx0,Gv=−1Revx+uy2vxvz+wy0,Hv=−1Rewx+uzwy+vz2wz0.

Above, *λ* is the pseudo-compressibility coefficient; *p* is pressure; *u*, *v*, and *w* are velocity components in the Cartesian coordinate system X, Y, and Z; *t* denotes physical time, while *τ* is pseudo-time; and *Re* is the Reynolds number. The term *q* associated with the pseudo-time is designed for an inter-iteration at each physical time step, which will vanish when the divergence of velocity is driven to zero to satisfy the equation of continuity. Reynolds number is defined as

(2) $$Re=\frac{U_{ref}L_{ref}}{\nu },\;\;$$

where *U_ref_* is a reference velocity, *L_ref_* is a reference length, and *ν* is the kinematic viscosity of air. The forewing mean chord length is used as the reference length. The mean wingtip velocity of the forewing is used as the reference velocity; *U_ref_* = *U_tip_* = ω*R*, where *R* is the span length and *ω* is the mean angular velocity of the flapping wing (*ω* = 2*Φf*, where *Φ* is the wing positional angle amplitude and *f* is the flapping frequency). When we solve the Navier–Stokes equations for a wing block, the aerodynamic forces exerted on the wing are evaluated by a sum of inviscid and viscous flux over the wing surface as

(3) $$F_{aero}\left(F_{x},F_{y},F_{z}\right)=-\sum_{i}^{n}\left({Flux}_{invis}+{Flux}_{vis}\right),$$

where *n* denotes the cell number on the surface of the wing. This CFD solver has been validated by comparison with some experimental studies, including the case of the hawkmoth wing [24].

**Figure 4:**
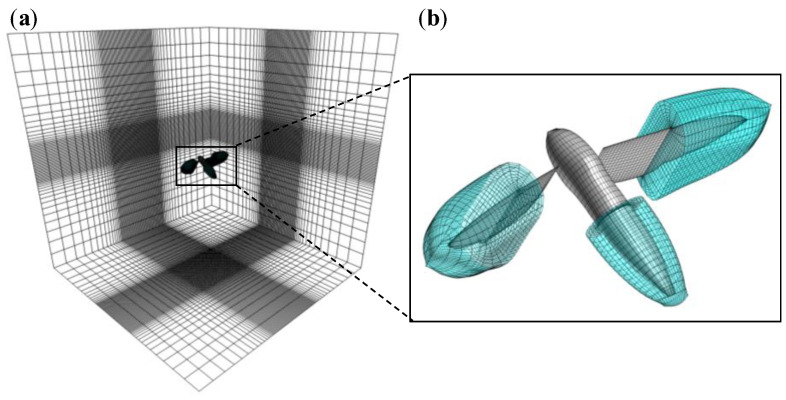
The computational domains: (**a**) global grid and (**b**) grids of a hawkmoth body and wings. Note that the gray and the blue colors represent the surface and the outermost grids.

In the present study, the computational domain was a Cartesian grid with 10*R* × 10*R* × 10*R* dimensions in which the body grid and the left- and right-wing grids (flyer blocks) were immersed (Figure 4). The Cartesian grid has two sub-regions: the clustering region, which has small uniform grid spacing, and the global region, which is gradually refined towards the center of the computational domain. The uniform grid spacing is set to 0.15 *Cm* in the model. The outer boundaries of the flyer blocks are immersed in the clustering region to prevent loss of accuracy due to the interpolation between the global block and flyer blocks. The numbers of grid points are the following: the global grid is 73 × 81 × 61; the body grid is 35 × 35 × 9; and the left- and right-wing grids are 45 × 45 × 15. The wing grids were clustered along the edges of the wing for higher resolution in the regions where strong shear flow is expected. The averaged grid size on the wing surface is approximately 0.045 *Cm* and 0.066 *Cm* in the chordwise and spanwise directions. 

### 2.3. Wing Kinematics Modifications for Hovering Equilibrium Conditions

For the perturbation analysis of 3 degree-of-freedom (DoF) flight dynamics and passive dynamic stability, the hovering equilibrium conditions must be achieved. In this study, the body and wings are assumed as rigid models without considering the deformations, and the center of mass is calculated under the constant density distribution of the body and wings. The results of the computational aerodynamic forces using the wing kinematic model constructed based on the filming experiment described previously show that there are differences from the hovering equilibrium condition (i.e., the state in which the cycle-averaged values of the aerodynamic forces *F_X_*, *F_Z_*, and the aerodynamic torque *T_Y_* are zero). Therefore, in this study, the following kinematic parameters were adjusted based on the previous studies [25,26] to achieve the hovering equilibrium conditions in 3 DoF flight dynamics—*F_X_*: the amplitude center of the feathering angle; *F_Z_*: flapping frequency; and *T_Y_*: the center of mass in the Z-axis. Note that many insects have been observed to behave in a similar manner when their wings are damaged. For example, they often increase their flapping frequency to compensate for the reduction in wing area for the generation of lift (i.e., hawkmoth [27], damselfly [28], honeybee [29]). Table 2 shows the adjusted parameters and the resultant cycle-averaged aerodynamic forces and torques. Note that the measured value of the center of mass is set to zero as the initial position and that the values in parentheses are the deviation from the experimental results. The forewing model has a smaller wing area than the full-winged model, so the flapping frequency was increased to compensate for the lift force. The Reynolds number—*Re* of each tuning model was calculated by Equation (2)—is *Re* = 4392 for the full-winged model and *Re* = 3350 for the forewing model. Both models are on the order of 10^3^, and no significant difference in aerodynamic properties is expected.

### 2.4. Numerical Model for Flight Dynamics: Equations of 3 DoF Motions

In the present study, we treat the flyer as a bilaterally symmetric rigid model and investigate its 3 DoF motions in the longitudinal direction. In actual insect flight, the flapping motion of the wings causes a shift of the center of mass; however, because of its high frequency and the light mass of the wings in a hawkmoth, about 5 to 6% of the body [30], the morphological effect caused by the flapping motion is negligible compared with that of the body. Therefore, we assume that the center of mass is fixed and the moment of inertia of the body is constant regarding the body-fixed coordinate system for the treatment of the rigid body model [31]. Then, a single rigid body dynamic model can be reconstructed based on the Newton–Euler equations of 3 DoF motions as a set of three coupled nonlinear ordinary differential equations, such as

(4) $${\dot{u}}_{b}=-q_{b}\cdot w_{b}+gsin\theta +\frac{F_{xb}}{m},\;{\dot{w}}_{b}=q_{b}\cdot u_{b}-gcos\theta +\frac{F_{zb}}{m},\;{\dot{q}}_{b}=\frac{T_{yb}}{I_{yy}},$$

where *F_xb_* and *F_zb_* denote the aerodynamic forces acting along the x_b_- and z_b_- axes, respectively; *T_yb_* is the aerodynamic pitching torque; *θ* is the pitch angle; *u_b_* and *w_b_* are the two components of the translational velocity of the body; *q_b_* is the angular velocity of the body; and *I_yy_* is the moment of inertia about the pitch axis of the body. Note that the translational and angular velocities of the body(*u_b_*, *w_b_*, *q_b_*) are defined as three state variables that describe the 3 DoF motions in the body-fixed coordinate system. Body mass, m, moment of inertia, *I_yy_*, and gravitational acceleration, *g* = 9.8 m/s^2^, are assumed to be constant.

### 2.5. Nonlinear Stability Analysis Based on the Perturbation Theory

Gao et al. performed the passive dynamic stability analysis with a relatively small perturbation (i.e., the magnitudes of the vertical and horizontal disturbances were less than 5% of the cycle-averaged flapping velocity of the flyer) by applying the first-order approximation based on the assumption that the aerodynamic forces and torques vary linearly in the directions of the disturbances [32]. However, in the aerial environment of a flying insect, there are usually sharp velocity gradients and wing gusts and, hence, large disturbances [33]. Some studies have been conducted to analyze the nonlinear flight dynamics of hovering model insects by numerically solving the equations of motion coupled with Navier–Stokes equations to simulate large disturbance motions [34], but these studies are computationally expensive. Therefore, in the present study, considering that the aerodynamic forces are on the scale of the square of the reference velocity, the second-order approximation is implemented to express the differences on the aerodynamic forces and torque with the perturbations. The construction is as follows.

First, a function representing aerodynamic forces and torque is defined and constructed in the form of a Fourier series, considering four conditions—one equilibrium condition and three unidirectional perturbation conditions. Under equilibrium conditions, time-varying aerodynamic forces and moments based on the CFD model can be decomposed into a Fourier series for a complete wing beat cycle [35], in the form of

(5) $$F(t)=\sum_{n=0}^{h}\left(a_{n}\cos\left(nkt\right)+b_{n}\sin\left(nkt\right)\right),\;\;$$

where *k* is the reduced frequency; *a_n_* and *b_n_* are Fourier series coefficients. Note that the coefficient, *a*_0_ represents the cycle-average aerodynamic force or torque over a single flapping cycle. The higher the harmonic *h*, the better this function fits the original value. In the present study, the tenth-order Fourier series (*h* = 10) was found to be sufficient for reproducing all the waveforms of the aerodynamic forces and torque generated by the CFD solver.

Second, considering that the perturbation conditions are achieved by specifying the deviation of the translational velocity and angular velocity of the body, the function in Equation (5) should change with both time t and time-varying state variables. Therefore, it is necessary to construct a system function in terms of these four variables (*t*, *u_b_*, *w_b_*, *q_b_*) to solve the equations of motion in Equation (4) while updating the time-varying aerodynamic forces and torque. To construct this system function, a CFD solver was utilized to obtain the aerodynamic forces and torque under the perturbation conditions in three directions. For the horizontal direction, the dimensionless translational velocity was given as eight different perturbation conditions in the range −1.0 < *u* < 1.0, as well as *w* for the vertical direction. For the pitch direction, the dimensionless angular velocity was given as six perturbations in the range −0.05 < *q* < 0.05. Note that *u* and *w* are the dimensionless inflow velocities in the X- and Z-axes. Using these results, the aerodynamic forces and torque under the perturbation conditions are approximated by introducing a quadratic function, such as

(6) $$\left[\begin{array}{c} F_{x}\\ F_{z}\\ T_{y} \end{array}\right]=\left[\begin{array}{c} F_{xe}\\ F_{ze}\\ T_{ye} \end{array}\right]+\left[\begin{array}{ccc} C_{F_{x},u} & C_{F_{x},w} & C_{F_{X},q}\\ C_{F_{z},u} & C_{F_{z},w} & C_{F_{z},q}\\ C_{T_{y},u} & C_{T_{y},w} & C_{T_{y},q} \end{array}\right]\cdot \left[\begin{array}{c} u\\ w\\ q \end{array}\right]+\left[\begin{array}{ccc} D_{F_{x},u} & D_{F_{x},w} & D_{F_{X},q}\\ D_{F_{z},u} & D_{F_{z},w} & D_{F_{z},q}\\ D_{T_{y},u} & D_{T_{y},w} & D_{T_{y},q} \end{array}\right]\cdot \left[\begin{array}{c} u^{2}\\ w^{2}\\ q^{2} \end{array}\right],$$

where

$$\left[\begin{array}{c} F_{xe}\\ F_{ze}\\ T_{ye} \end{array}\right]=\sum_{n=0}^{10}\left(a_{i,n}\cos\left(n\omega t\right)+b_{i,n}\sin\left(n\omega t\right)\right),\;C_{i,j}=\sum_{n=0}^{10}\left(c_{i,j,n}\cos\left(n\omega t\right)+d_{i,j,n}\sin\left(n\omega t\right)\right),\;\;D_{i,j}=\sum_{n=0}^{10}\left(e_{i,j,n}\cos\left(n\omega t\right)+f_{i,j,n}\sin\left(n\omega t\right)\right).$$

Above, the forces and torque (*F_xe_*, *F_ze_*, *T_ye_*) are obtained under the trimmed flight condition without perturbation (see Section 2.3); *ω* is the angular frequency of the wing beat; *a*~*f* are Fourier series coefficients; the subscript of *i* denotes the aerodynamic force and torque components and *j* denotes the perturbation components. 

At this stage, the solution of Equation (6) is transformed to the body-fixed coordinate system, yielding *F_xb_*, *F_zb_*, and *T_yb_* which can be utilized for Equation (4). Finally, it was possible to simulate the state variables under the perturbation conditions by performing the time integration while inserting the perturbations obtained from Equation (4) into Equation (6) at each time step.

## 3. Results and Discussion

### 3.1. Aerodynamic Performance of a Hawkmoth with/without Hindwings

**Figure 5:**
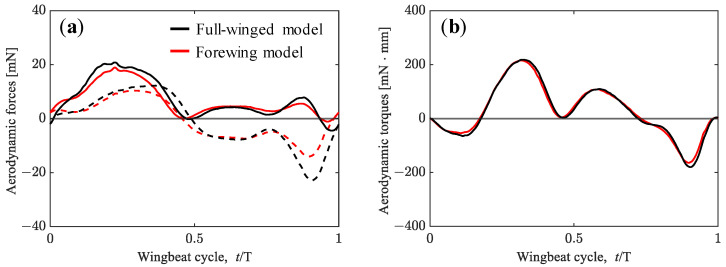
(**a**) Vertical (solid lines) and horizontal (dashed lines) forces acting on the hawkmoth with (black lines)/without (red lines) hindwings by adopting the measured wing kinematics. (**b**) Aerodynamic torque.

First, to clarify the effects of the hindwings on the aerodynamic performance of the hovering hawkmoth, we performed the numerical simulation of the full-winged and forewing models by adopting the measured wing kinematics (Figure 3). The difference between these two models here is the wing area; the wing area of the forewing model is about 73% of that of the full-winged model. Regarding force production, it is known that the force generated by a flapping or revolving wing is proportional to the second moment of the wing area [36]. In the present study, the second moment of the wing area can be calculated as

(7) $$S=\int_{0}^{R}C_{m}\left(r\right)r^{2}dr,\;\;$$

where r denotes the spanwise location of the local wing element. The second moment of the wing area of the forewing model results in 93% of that of the full-winged model. The vertical and horizontal aerodynamic forces and pitching torque during a wingbeat cycle are depicted in Figure 5, and the cycle-averaged aerodynamic forces, wing area (refer again as in Table 1), and the second moment of wing area are summarized in Table 3. The values in parentheses for the forewing model indicate the percentages compared with the full-winged model.

**Figure 6:**
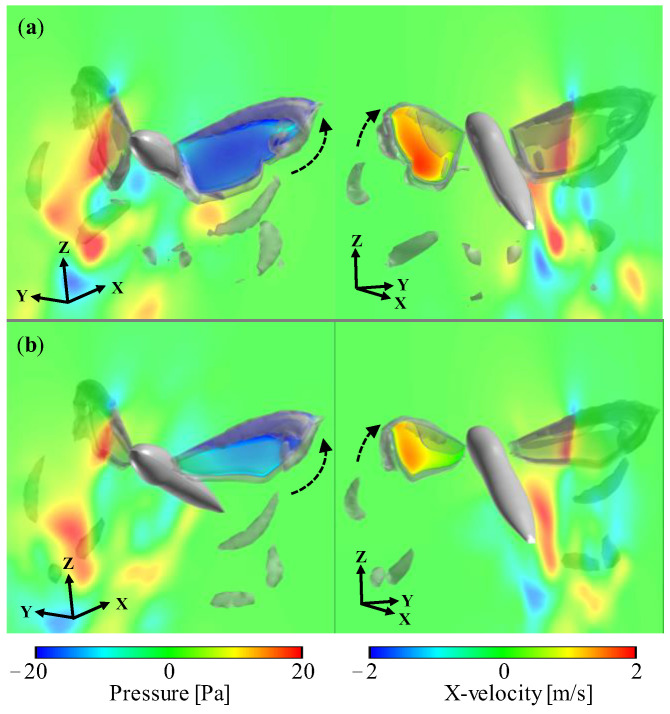
Pressure distribution on the upper/lower left wing surface and X-velocity field in the XZ-plane of (**a**) full-winged and (**b**) forewing models visualized from two different views. The gray smoke-like object is the iso-surface of *Q*-criterion at 3.0 × 10^5^ [s^−2^]. Dashed arrows indicate the direction of wing motion.

The considerable difference in aerodynamic forces between the full-winged and forewing models can be seen in the timing around *t*/*T* = 0.9. At this timing, the wings are flapping with a geometric angle of attack close to 90 degrees, generating the large positive pressure region on the upper surface and the large negative pressure region on the lower surface, resulting in a large drag-based thrust (Figure 6). This thrust is greater in the full-winged model which has a longer chord length than the forewing model, and the positive velocity increase in the X-axis can be seen in the visualized X-velocity distributions around the wings. 

**Figure 7:**
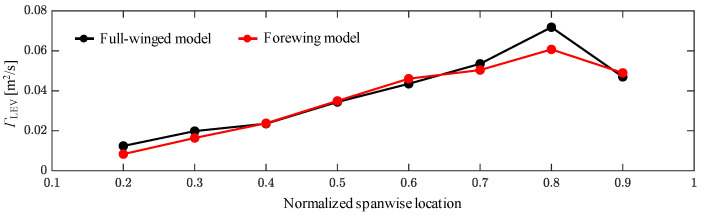
Spanwise distributions of LEV circulations at *t*/*T* = 0.2.

**Figure 8:**
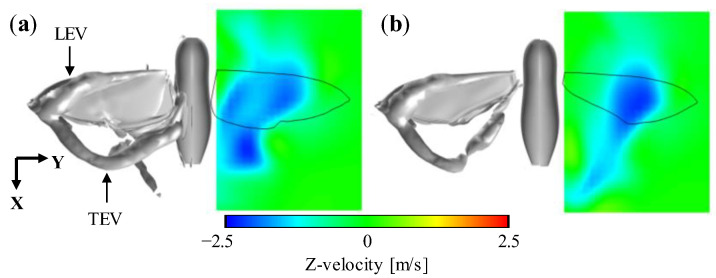
The iso-surface of *Q*-criterion at 3.7 × 10^5^ [s^−2^] and the vertical velocity distributions in the XY-plane near the lower wing surface for (**a**) full-winged and (**b**) forewing models.

In terms of lift force during downstroke, the full-winged model maintains a greater lift force than the forewing model at approximately a constant rate, and the average values of lift force during downstroke are 11.79 and 10.18 [mN], respectively. The delayed stall of the leading-edge vortex (LEV) on the wing surface during the flapping motion has been observed [3,37,38,39], and it can promote the lift force. The presence of the LEV on the wing surface creates a relatively low-pressure region, which increases the lift force. To clarify the differences in lift force in the present study, we quantified the strength (circulation) of the LEV at *t*/*T* = 0.2, where the maximum lift forces occur in both models. The LEV circulation was estimated by integrating the vorticity as follows.

(8) $$\Gamma_{LEV}=\iint_{S}^{}\omega \cdot dS,\;\;$$

where ω is the vorticity. Note that the area of integration, *dS*, is bound by the vorticity threshold for capturing the LEV (25% of the peak vorticity in this study) as in [40]. The spanwise distributions of the instantaneous LEV circulation at *t*/*T* = 0.2 are shown in Figure 7. In both the full-winged and forewing models, the LEV intensities increase linearly with almost the same slope from the wing root to 0.8*R* at the wingtip. Chen et al. adopted a revolving wing model of a hawkmoth and reported that LEV formation is almost the same when the hindwings are removed, and thus, the forewings can produce most of the aerodynamic forces [41], indicating that the results adopting the flapping kinematics in the present study show similar results for LEV formation. The decrease in the values at 0.9*R* is caused by the interference of the wing tip vortex. The only considerable difference occurs in the circulation at 0.8*R*, where the full-winged model shows about an 18% increase compared with the forewing model. Phillips et al. performed the experiments with simple rectangular wings and insect kinematics and reported more enhanced LEV circulation across the span with an increasing aspect ratio (AR) [10]. On the other hand, Harbig et al. reported in their numerical calculations of a revolving wing with a fruit fly model that the narrower width per unit span of a high-AR wing causes the LEV to interact with the trailing-edge vortex (TEV) from the lower side of the wing, reducing the circulation and, thus, the lift [42]. Comparing cicada hindwings with hindwing-less Drosophila, in which the cicada wings have two distinct trailing-edge shapes with a reduced wing tip area and an increased wing root area, clear differences in trailing-edge vortex formulation and stabilization of the flow in the wing span direction have been reported to improve lift [43]. The difference in TEV structure was also observed in the present study (Figure 8), where the TEV of the forewing model was more backwardly tilted along its trailing-edge shape around the wing root compared with the full-winged model, and it shed from the wing surface earlier than the full-winged model. The local difference in LEV intensity between the full-winged and forewing models when the area is reduced only on the wing root side, as in this study, may not be due to the effect of AR but rather to the difference in the trailing-edge vortex. In addition, the difference in the vertical velocity distributions (Figure 8) indicates that the downwash is more widely spread on the wing root side in the full-winged model than in the forewing model, resulting in greater lift near the wing root as well. Because the wingtip vortices in both models do not show significant differences, this may not be caused by a change in the relative angle of attack due to the downward velocity induced by the wingtip vortices, but rather, it is due to the fact that the TEV remains distinct near the trailing edge, as seen in Figure 8 and in the longer chord length in the full-winged model. It is also possible that the spread of downwash in the wing root region may enhance the stability of the LEV, as seen in the circulation at 0.8*R* [44,45,46].

In flapping flight, the cycle-averaged aerodynamic force-to-power ratio (*F*_Σ_/*P*) can be utilized for estimating the efficiency [47]. The aerodynamic power imparted to the air by each wing is defined as

(9) $$P=-\sum_{i}^{N}\left(F_{aero,i}\cdot v_{surf,i}\right),\;\;$$

where *N* denotes the number of elements on the surface of each wing; *F_aero_* is the aerodynamic force acting on each element; and *v_surf_* is the corresponding velocity of each element. The efficiencies of the left and right wings in generating lift force are 19.5% for the full-winged model and 22.8% for the forewing model, with the forewing model being slightly more efficient. Similarly, numerical results using a revolving hawkmoth wing model with/without hindwings reported improved aerodynamic efficiency of a forewing model [41]. In the comparison of the cycle-averaged values, 30% and 7% reductions in horizontal and vertical forces are observed due to the reduction in wing area. From the morphological parameters of the wings in Table 3, the reduction of the horizontal force in the forewing model is almost equal to the reduction ratio of the wing area (=27%), and the reduction of the vertical force is almost identical to the reduction of the second moment of the wing area (=7%). The cycle-averaged aerodynamic torque was slightly higher in the pitch-up direction for the full-winged model. This may be caused by the moving of the center of the air pressure closer to the center of mass in the forewing model. 

**Figure 9:**
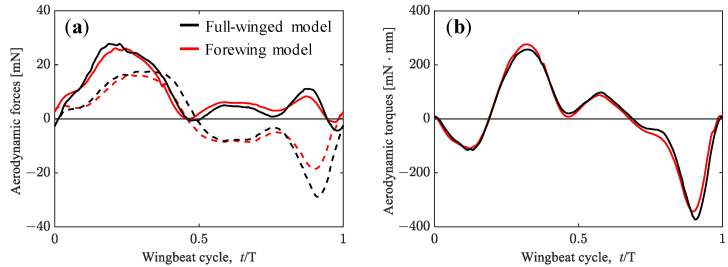
(**a**) Vertical (solid lines) and horizontal (dashed lines) forces acting on the hawkmoth with (black lines)/without (red lines) hindwings during the trimmed flight. (**b**) Aerodynamic torque.

The aerodynamic forces obtained from trimmed flight by adopting the tuned wing kinematics for the full-winged and forewing models are shown in Figure 9. The differences in the wing kinematics that affect the aerodynamic force generation in both models are the flapping frequency and the amplitude center of the feathering angle, which differ by 1.7 Hz and 1.2°, respectively (see Table 2). While the maximum lift and thrust increased with increasing flapping frequency in the tuned wing kinematic models, the waveforms of the aerodynamic forces and torque were similar to those of the measured wing kinematic models for both the full-winged and forewing models (Figure 5 vs. Figure 9). For the models with the tuned wing kinematics here, the cycle-averaged values of the aerodynamic horizontal force and the aerodynamic torque in the pitch direction were close to zero, and the vertical aerodynamic force is nearly balanced by its own weight, indicating that a state close to trimmed flight was achieved (Table 2).

### 3.2. Hovering Equilibrium Condition

**Figure 10:**
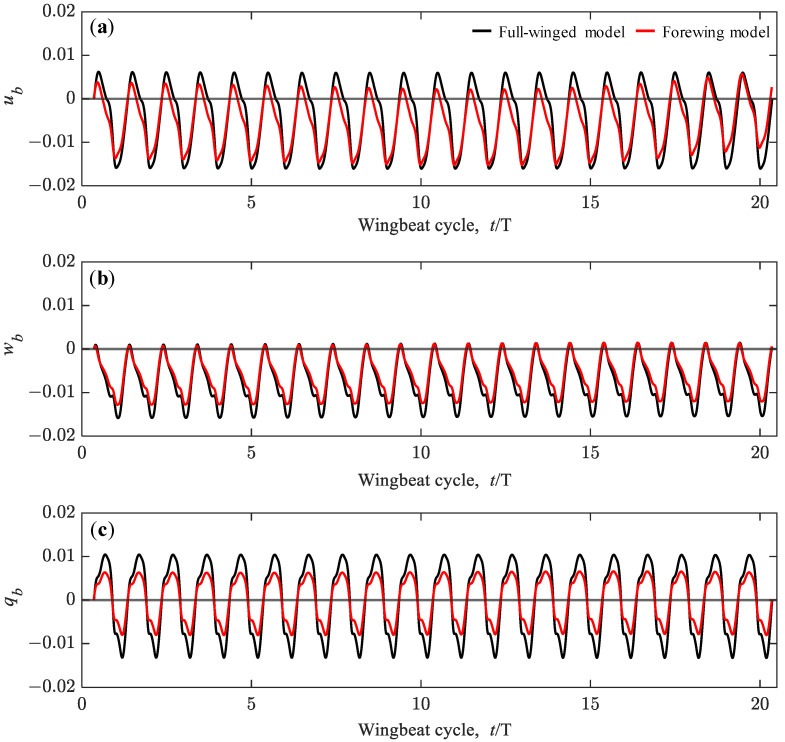
The time histories of the state variables of (**a**) *u_b_*, (**b**) *w_b_*, and (**c**) *q_b_* up to 20 wingbeat cycles under the no perturbation conditions. All the state variables are shown as dimensionless values.

As discussed in previous studies [32], the initial values of aerodynamic forces and torque in the flight dynamic analysis have a significant effect on the subsequent dynamic behavior, and thus, the initial time of the dynamic analysis must be adjusted to achieve a long-term hovering equilibrium condition in the absence of active control of the wing motion. As a result of the parameter study, appropriate initial times of *t*_0_ = 0.3574 and 0.3487 were obtained for the full-winged and forewing models. With these initial times, the equilibrium conditions are achieved up to 20 beat cycles and the time variation of the state variables *u_b_*, *w_b_* and *q_b_* oscillate in a range of −0.016 < *u_b_* < 0.006, −0.016 < *w_b_* < 0.001, and −0.013 < *q_b_* < 0.010 for the full-winged model and −0.015 < *u_b_* < 0.006, −0.013 < *w_b_* < 0.001, and −0.008 < *q_b_* < 0.007 for the forewing model (Figure 10). In the present study, these equilibrium conditions were defined as stable hovering flight, and then the dynamic analysis was performed by adding the various perturbations.

### 3.3. Passive Dynamic Stability with Relative Small Perturbations 

**Figure 11:**
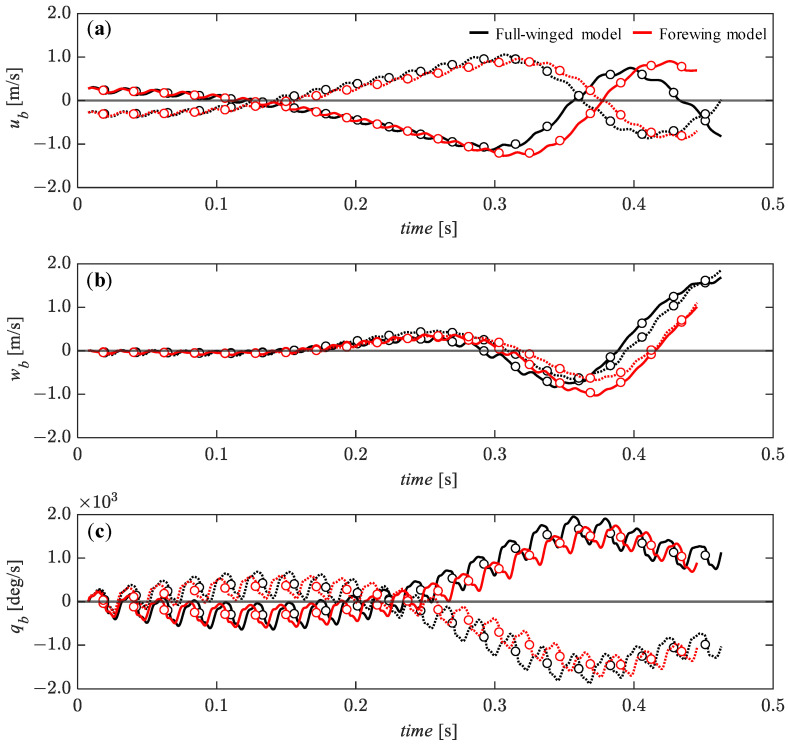
The time histories of the state variables of (**a**) *u_b_*, (**b**) *w_b_*, and (**c**) *q_b_* under the initial conditions of *u_b_* = 0.28 (solid lines) and *u_b_* = −0.28 (dashed lines) [m/s] up to 20 wingbeat cycles. Circle markers represent the cycle-averaged values at each wingbeat cycle.

In this section, the dynamic analysis was performed under relatively small perturbations—5% of the reference velocity for the *u_b_* and *w_b_* components and 0.5% of the reference angular velocity for the *q_b_*. Figure 11 presents the time histories of the state variables under the initial conditions of *u_b_* = 0.05 (solid line) and *u_b_* = −0.05 (dashed line). It can be seen that the x_b_-axial velocity component (*u_b_*) converges monotonically and gradually to an equilibrium state up to about *t* = 0.15 [s] (Figure 11a) while the z_b_-axial velocity component (*w_b_*) remains in an equilibrium condition (Figure 11b). The pitch angular velocity, *q_b_* tends to gradually diverge up to this time, but after *t* = 0.15 [s], it gradually converges to an equilibrium state until about *t* = 0.25 [s] (Figure 11c) while the *u_b_* and *w_b_* tend to diverge during this time. The pitch-up motion (*q_b_* is positive) due to the forward perturbation (*u_b_* = −0.05) and the pitch-down motion (*q_b_* is negative) due to the backward perturbation (*u_b_* = 0.05) during the first few flapping cycles are similar in trend to the passive response of Drosophila with a high aspect ratio wing [32]. This passive response is likely to promote convergence because the pitch-up/down motion leads to a modulation of increased backward/forward aerodynamic forces [48], which can passively reduce the forward/backward deviation. There is no significant difference depending on the presence or absence of hindwings, but the *u_b_* and *w_b_* recover to a transient equilibrium state slightly earlier in the full-winged model than in the forewing model.

**Figure 12:**
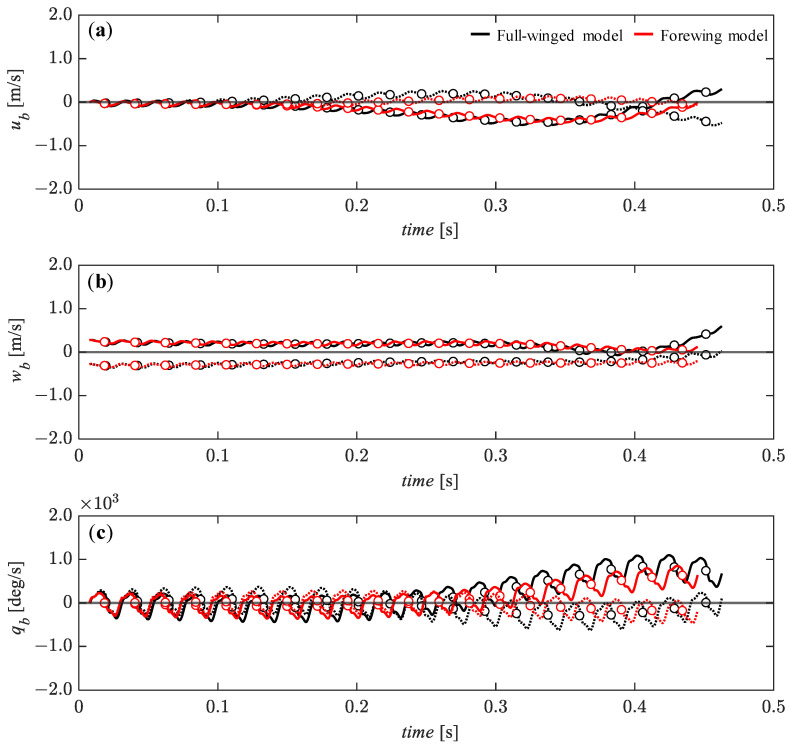
The time histories of the state variables of (**a**) *u*_b_**, (**b**) *w*_b_**, and (**c**) *q*_b_** under the initial conditions of *w_b_* = 0.28 (solid lines) and *w_b_* = −0.28 (dashed lines) [m/s] up to 20 wingbeat cycles. Circle markers represent the cycle-averaged values at each wingbeat cycle.

For the vertical initial perturbations of *w_b_* = 0.05 (solid line) and *w_b_* = −0.05 (dashed line), it can be seen that the overall transitions of the state variables are more stable than for the horizontal perturbations (Figure 11 vs. Figure 12). Although there is a slight divergence trend in the *u_b_* (Figure 12a), all state variables remain stable up to about *t* = 0.25 [s]. In particular, for the w_b_, the passive restoring force acts steadily and monotonically up to about *t* = 0.4 [s] (Figure 12b). After *t* = 0.25 [s], the *u_b_* and the pitch angular velocity, *q_b_* are more stable for the downward perturbation than for the upward perturbation, with the same trend for both full-winged and forewing models. The difference between the full-winged and the forewing models can be seen in the pitch angular velocity after *t* = 0.3 [s] (Figure 12c). The forewing model is able to delay the divergence trend for the upward perturbation compared with the full-winged model. The stable behavior of the translational velocity and the development of the pitch instability in response to the vertical perturbations in both models are similar to those observed in the analysis of nonlinear flight dynamics with the fully coupled Navier–Stokes equation and the equation of motion [34].

**Figure 13:**
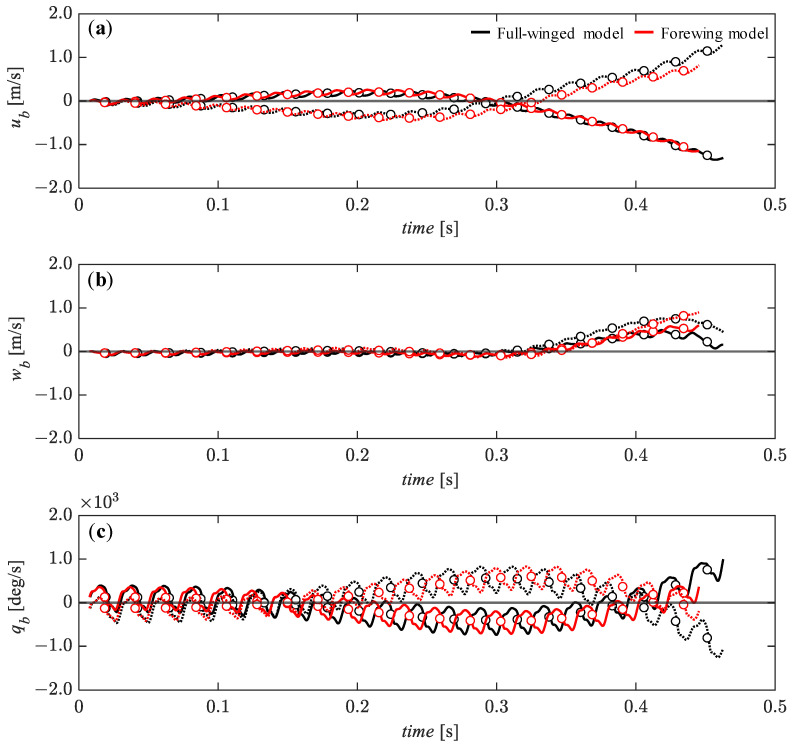
The time histories of the state variables of (**a**) *u_b_*, (**b**) *w_b_*, and (**c**) *q_b_* under the initial conditions of *q_b_* = 1.28 × 10^2^ (solid lines) and *q_b_* = −1.28 × 10^2^ (dashed lines) [deg/s] up to 20 wingbeat cycles. Circle markers represent the cycle-averaged values at each wingbeat cycle.

With respect to the pitch perturbations, there is a slightly more stable transition for the *u_b_* around *t* = 0.1 to 0.3 [s] for the pitch-up (solid lines) perturbation compared with the pitch-down (dashed line) perturbation (Figure 13a), and this trend is also observed for the pitch angular velocity, *q_b_* around *t* = 0.2 to 0.4 [s] (Figure 13c). The *w_b_* is less affected by the pitch perturbation and remains in the equilibrium state until about *t* = 0.3 [s] (Figure 13b) when the *u_b_* starts to diverge significantly at this timing. Although there is no significant difference between the full-winged and the forewing models, the *u_b_* and *w_b_* around *t* = 0.3 to 0.4 [s] under the pitch-down perturbation have a slightly smaller slope in the positive direction for the forewing model than for the full-winged model, indicating that the forewing model is less affected by the pitch perturbations.

### 3.4. Passive Dynamic Stability with Relatively Large Perturbations

**Figure 14:**
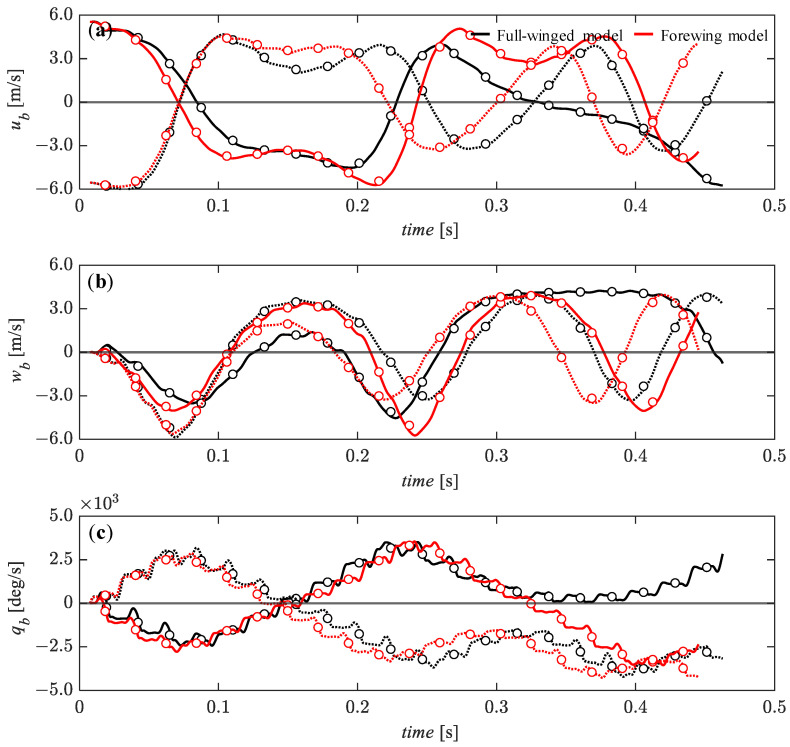
The time histories of the state variables of (**a**) *u_b_*, (**b**) *w_b_*, and (**c**) *q_b_* under the initial conditions of *u_b_* = 5.54 (solid lines) and *u_b_* = −5.54 (dashed lines) [m/s] up to 20 wingbeat cycles. Circle markers represent the cycle-averaged values at each wingbeat cycle.

Here, we present the response of the state variables to the large perturbations—100% of the reference velocity for the *u_b_* and *w_b_* components and 5% of the reference angular velocity for the *q_b_*. For the forward and backward perturbations of the *u_b_*, the response of the *u_b_* tends to converge during the first few flapping cycles as in the case of a small perturbation of the *u_b_* (Figure 11a and Figure 14a), while the *w_b_* and pitch angular velocity, *q_b_* diverge significantly during this period (Figure 14b,c). Note that the response of the pitch angular velocity, *q_b_* during the first three cycles acts to passively reduce the forward/backward deviation, as in the case of the small perturbation of the *u_b_*. Thereafter, the large monotonic oscillations tend to begin in all state variables. After *t* = 0.32 [s], the full-winged and forewing models show differences in response to the backward perturbation with the forewing model continuing the large oscillations in all state variables while the full-winged model shows different behavior. The full-winged model shows stable behavior with respect to the *u_b_* and the pitch angular velocity, *q_b_* maintaining an almost equilibrium state for a short period.

**Figure 15:**
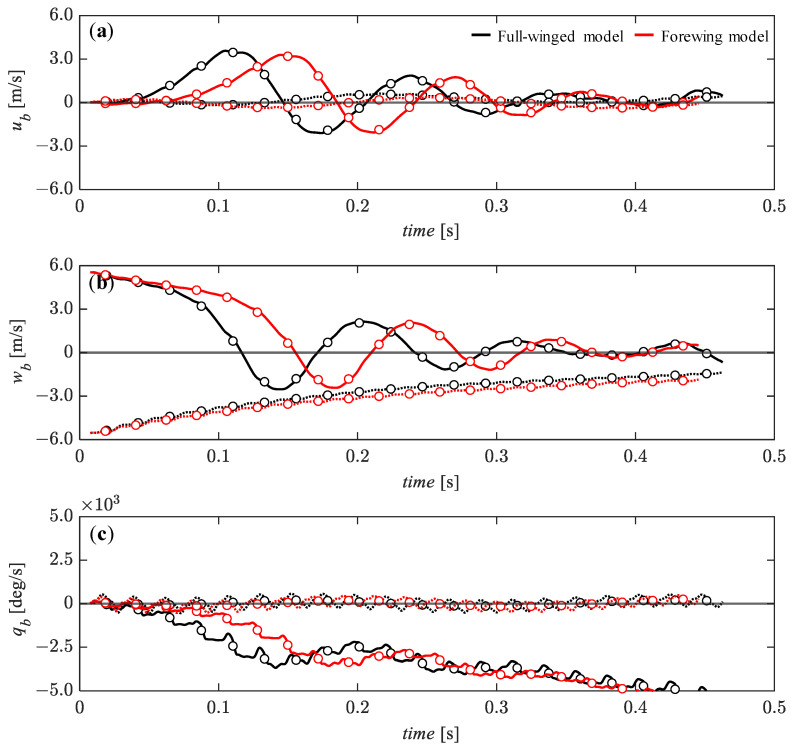
The time histories of the state variables of (**a**) *u_b_*, (**b**) *w_b_*, and (**c**) *q_b_* under the initial conditions of *w_b_* = 5.54 (solid lines) and *w_b_* = −5.54 (dashed lines) [m/s] up to 20 wingbeat cycles. Circle markers represent the cycle-averaged values at each wingbeat cycle.

The most stable passive restoring forces in the present study were found in the response of the *u_b_* and *w_b_* to large vertical perturbations (Figure 15a,b). For the upward perturbation (solid lines), the *u_b_* initially diverges in a positive direction but then becomes a damped oscillation, similar to the response of the *w_b_*; finally, both values recover to an equilibrium state in about *t* = 0.4 [s]. For the downward perturbation (dashed lines), the *u_b_* is almost unaffected and remains in the equilibrium state, as in the case of the small perturbation (Figure 12a and Figure 15a), while the *w_b_* shifts monotonically and gradually from the initial perturbation values to an equilibrium state (Figure 15b). The pitch angular velocity, *q_b_* has little effect for the downward perturbation but is significantly shifted away from the equilibrium state for the upward perturbation (Figure 15c). This clear difference in the pitch instability due to the directions of the large vertical perturbations is also observed in the previous study [34]. Under the large vertical perturbation, the differences in the response of the state variables between the full-winged and forewing models were observed that were not seen under the small perturbation. In the full-winged model, the *u_b_*, the *w_b_*, and the pitch angular velocity, *q_b_* are all more sensitive to the upward perturbation than in the forewing model, indicating that the presence of hindwings has a greater effect in this case.

**Figure 16:**
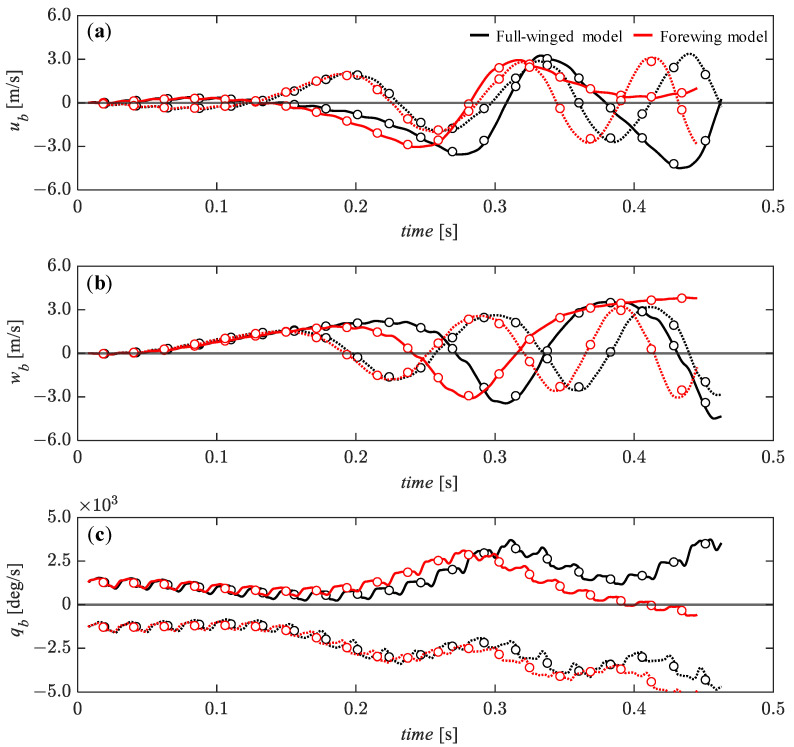
The time histories of the state variables of (**a**) *u_b_*, (**b**) *w_b_*, and (**c**) *q_b_* under the initial conditions of *q_b_* = 1.28 × 10^3^ (solid lines) and *q_b_* = −1.28 × 10^3^ (dashed lines) [deg/s] up to 20 wingbeat cycles. Circle markers represent the cycle-averaged values at each wingbeat cycle.

With respect to the pitch perturbation, the *u_b_* and the pitch angular velocity, *q_b_* tend to converge to the equilibrium state until about *t* = 0.1 [s], but thereafter all state variables oscillate significantly by increasing the *w_b_*, and the amplitude tends to increase with time. As seen in the response to the large perturbation in the *u_b_*, after *t* = 0.32 [s], the full-winged and forewing models show the difference in response to this pitch perturbation (Figure 14 and Figure 16). The full-winged model continues to oscillate significantly for all state variables while the forewing model shows the behavior that breaks the periodic motion at this time. The forewing model shows a gradual convergence to the equilibrium state with respect to *u_b_* and pitch angular velocity, *q_b_* until about *t* = 0.4 [s]. 

These behaviors show that the full-winged model is partially stable for the horizontal perturbation while the forewing model is partially stable for the pitch perturbation, indicating that the hindwing can act for better or worse depending on the direction of the perturbation.

### 3.5. Effect of Hindwings on Passive Dynamic Stability

The results of the passive dynamic stability analysis with the hawkmoth in the present study showed that for some perturbations, the state variables converged to the equilibrium state condition as reported in the study on the longitudinal dynamic stability of a hawkmoth adopting a linear time-invariant dynamic model [34], but for other perturbations, an inherent instability was observed as reported for the desert locust and the bumblebee in the open-loop conditions with a linear theory [15,49]. There are some small differences in flight behavior with/without hindwings in the present study, but no clear trend was observed. In nature, insects suffer wing damage throughout their lives [50], but even if they have lost or damaged their hindwings, most of them can still fly, so the intrinsic influence of hindwings on passive flight stability may be small. However, in terms of flight maneuverability, in the flights of moths and butterflies (Lymantria dispar and Pieris rapae) with severed hindwings, which have a larger ratio of hindwings to total wing area than the model in this study, a reduction in flight maneuverability in terms of linear and turning acceleration has been reported as a result of hindwing resection [20]. In the escape flights of moths with varying hindwing shapes in response to bat predation, it was reported that species with longer hindwings in the chordwise direction were more successful in deflecting predation by bats [51]. Flying insects are known to adopt flight strategies that actively modify wing kinematic parameters, such as flapping frequency, flapping amplitude, and stroke plane angle [27,28,29], when wing area is reduced due to wing damage. In terms of passive flight stability, it has been reported that wing flexibility can improve flight stability [52]. It should be noted that a comprehensive evaluation of the unique impact of hindwings on flight stability in natural insects may be possible by considering active changes in wing kinematics and passive wing deformation, which were not considered in this study.

The delay in controlling active wing motion in insects is estimated to be several flapping cycles [14], and the results of passive flight behavior in this study for the long flapping cycles may not necessarily be related to the flight behavior of actual insects; however, this study does play a great role in considering the wing design in FMAVs. In particular, the presented results may be useful to guide the design of wings in terms of whether to adopt wings with a high aspect ratio to improve flight maneuverability, or wings with a small aspect ratio and a large wing root area to achieve a high passive response and damped vibration during the first few flapping cycles to perturbations.

## 4. Conclusions

In the present study, we investigated the aerodynamic effects and passive dynamic stability with/without hindwings in the hawkmoth, *Agrius convoluvuli*. In terms of aerodynamic effects, it was suggested that the hindwings may contribute to the enhancement of partial LEV circulation during downstrokes due to the stable TEV of the hindwings. An increase in downwash at the hindwings was also observed while the hindwings have a slight negative effect on the efficiency of lift generation. For thrust generation, a significant increase was observed in the region of the hindwings during upstrokes. In terms of passive dynamic stability, the results showed that the presence or absence of the hindwings did not show a significant trend, but the hindwings may contribute to improving flight stability depending on the direction of any perturbations. The results obtained in this study may be useful for the integrated development of wing geometry and flight control for FMAVs.

## Figures and Tables

**Table 1:** Morphological parameters for full-winged and forewing models.

	Full-Winged Model	Forewing Model
Total mass [mg]	956	946
Wing area [mm^2^]	443	325
Mean chord length, *C_m_* [mm]	12.39	9.10
Wing length, *R* [mm]	35.76	
Stroke plane angle [deg]	36.36	
Body length [mm]	41.70	
Body angle, *β* [deg]	36.36	

**Table 2:** Tuning parameters and resultant forces for full-winged and forewing models.

	Measurement Model	Full-Winged Model	Forewing Model
Amplitude center of the feathering angle [deg]	7.36	11.86 (+4.5)	10.66 (+3.3)
Flapping frequency, *f* [Hz]	39.14	44.00 (+4.86)	45.70 (+6.56)
Center of mass in Z-axis [mm]	0	−5.29	−6.66
Cycle-averaged horizontal force, *F*x [mN]	−1.13	−0.0019	0.012
Cycle-averaged vertical force, *F*z [mN]	7.28	9.35	9.30
Total weight [mN]	9.38	9.38	9.28
Cycle-averaged pitching torque, *T*y [mN·mm]	34.21	−0.011	0.017

**Table 3:** The cycle-averaged forces, torque, and the wing parameters.

	Full-Winged Model	Forewing Model
Cycle-averaged horizontal force, *F*x [mN]	−1.13	−0.80 (70%)
Cycle-averaged vertical force, *F*z [mN]	7.28	6.73 (93%)
Cycle-averaged pitching torque, *T*y [mN·mm]	34.21	28.53 (85%)
Wing area [mm^2^]	443	325 (73%)
Second moment of wing area [mm^4^]	1.41 × 10^−7^	1.31 × 10^−7^ (93%)

## Data Availability

The data generated and/or analyzed as well as the sources code during the current study are not publicly available due to their use in an undergoing project but could be available from the corresponding author upon reasonable request.
